# The DANTE trial protocol: a randomised phase III trial to evaluate the Duration of ANti-PD-1 monoclonal antibody Treatment in patients with metastatic mElanoma

**DOI:** 10.1186/s12885-021-08509-w

**Published:** 2021-07-01

**Authors:** Oliver Coen, Pippa Corrie, Helen Marshall, Ruth Plummer, Christian Ottensmeier, Jane Hook, Sue Bell, Gurdeep S. Sagoo, David Meads, Janine Bestall, Galina Velikova, Ferdia A. Gallagher, Alexandra Smith, Helen Howard, Ellen Mason, Eszter Katona, Shobha Silva, Michelle Collinson, Simon Rodwell, Sarah Danson

**Affiliations:** 1 Leeds Teaching Hospitals NHS Trust, Leeds, UK; 2 Cambridge University Hospitals NHS Foundation Trust, Cambridge, UK; 3 University of Leeds, Leeds, UK; 4 Clinical Trials Research Unit, Leeds Institute of Clinical Trials Research, Leeds, UK; 5 Newcastle University, Newcastle upon Tyne, UK; 6 University of Southampton, Southampton, UK; 7 Leeds Institute of Medical Research at St James’s University Hospital, Leeds, UK; 8 University of Cambridge, Cambridge, UK; 9 Sheffield Teaching Hospitals NHS Foundation Trust , Sheffield, UK; 10 University of Sheffield, Sheffield, UK; 11 Melanoma Focus, Cambridge, UK

**Keywords:** Immunotherapy, Checkpoint inhibitor, Anti-PD-1, Metastatic melanoma, Schedule, Efficacy, Safety, Quality of life

## Abstract

**Background:**

Immunotherapy is revolutionising the treatment of patients diagnosed with melanoma and other cancers. The first immune checkpoint inhibitor, ipilimumab (targeting cytotoxic T-lymphocyte-associated antigen 4 (CTLA-4)), showed a survival advantage over standard chemotherapy. Subsequently the anti-programmed cell death protein 1 (PD-1) antibodies, nivolumab and pembrolizumab were shown to be more effective than ipilimumab. Ipilimumab combined with nivolumab gives an incremental gain in overall survival compared with nivolumab alone but increases the risk of severe, potentially life-threatening toxicities. In contrast to ipilimumab monotherapy, anti-PD-1 antibodies are licensed to be continued until disease progression. Follow-up of patients recruited to the first trials evaluating 2 years of pembrolizumab showed that three-quarters of responding patients continue responding after stopping treatment. Suggestive of early response, we hypothesised that continuing anti-PD-1 treatment beyond 1 year in progression-free patients may be unnecessary and so designed the DANTE trial.

**Methods:**

DANTE is a multicentre, randomised, phase III, non-inferiority trial to evaluate the duration of anti-PD-1 therapy in patients with metastatic (unresectable stage III and stage IV) melanoma. It uses a two-stage recruitment strategy, registering patients before they complete 1 year of first-line anti-PD-1 +/− CTLA-4 therapy and randomising eligible patients who have received 12 months of treatment and are progression-free at 1 year. At randomisation, 1208 patients are assigned (1:1) to either 1) continue anti-PD-1 treatment until disease progression/ unacceptable toxicity/ for at least 2 years in the absence of disease progression/ unacceptable toxicity or 2) to stop treatment. Randomisation stratifies for baseline prognostic factors. The primary outcome is progression-free survival at 3, 6, 9 and 12 months and then, 6-monthly for up to 4-years. Secondary outcomes collected at all timepoints include overall survival, response-rate and duration and safety, with quality of life and cost-effectiveness outcomes collected 3-monthly for up to 18-months. Sub-studies include a qualitative analysis of patient acceptance of randomisation and sample collection to inform future translational studies into response/ toxicity biomarkers.

**Discussion:**

DANTE is a unique prospective trial investigating the optimal duration of anti-PD-1 therapy in metastatic melanoma patients. Outcomes will inform future use of these high burden drugs.

**Trial registration:**

ISRCTN15837212, 31 July 2018.

**Supplementary Information:**

The online version contains supplementary material available at 10.1186/s12885-021-08509-w.

## Background

Melanoma is the most aggressive form of skin cancer. For most patients diagnosed with primary melanoma, surgical excision alone is often sufficient with 1-year of adjuvant systemic therapy reserved for higher risk patients. Systemic therapy is offered to those patients diagnosed with metastastic (unresectable stage III or stage IV) disease. Until 2011, median survival was very poor at around 8 months [1]. In the last decade, median overall survival has increased now to around 3 years, due to the introduction of 2 classes of systemic anticancer agents: immune checkpoint inhibitors [2] and, in selected *BRAF*-mutant patients, mitogen activated protein (MAP) kinase pathway inhibitors [3].

Immune checkpoint inhibitors are now standard practice across multiple tumour sites including melanoma [4], lung [5], head and neck [6] and urological cancers [7,8]. In melanoma, therapeutic targets include the T cell receptors, cytotoxic T-lymphocyte-associated antigen 4 (CTLA-4) and programmed cell death protein 1 (PD-1) [9].

Ipilimumab, directed against CTLA-4, was the first immune checkpoint inhibitor to show improved overall survival for patients with metastatic melanoma in both the first-line [2] and second-line [10] setting. Subsequent trials demonstrated greater survival benefit from PD-1 blockade using the antibodies pembrolizumab [11] or nivolumab [12]. The CheckMate 067 trial compared ipilimumab combined with nivolumab for 12 weeks followed by nivolumab maintenance with nivolumab alone and with ipilimumab only and demonstrated 5 year overall survival of 52% for combined therapy, 44% for nivolumab monotherapy and 26% for ipilimumab after minimum follow up of 60 months [13]. In contrast to ipilimumab, which is given as 4 × 3 week infusions over 12 weeks, both pembrolizumab and nivolumab are licensed to continue regular infusions for as long as there is clinical benefit or until unacceptable toxicity. Five year outcomes of patients recruited to the KEYNOTE-001 trial [14] showed that while progression-free survival (PFS) was around 8 months, 29% of patients first treated with pembrolizumab were progression free at 5 years. Immune-checkpoint inhibitors have complex immune-related side effects, which range from being mild to potentially life threatening or life-changing. Onset most likely occurs early however late-onset toxicity (beyond 12 months) has previously been reported in 30% of patients completing a minimum treatment duration of 12 months [15]. The incidence of toxicity varies with regimen, with treatment related grade 3–4 adverse events (AE) occurring in 23% of nivolumab-treated, 28% of ipilimumab–treated and 59% of ipilimumab-nivolumab-treated patients [13]. Deaths are relatively rare [13].

In the UK, in line with current NICE guidance which covers England, both pembrolizumab [16] and nivolumab [17] are licensed monotherapies for first-line treatment of patients with metastatic melanoma. Ipilimumab-nivolumab is also licensed [18] and while there are no clear specifications as to how to select between anti-PD-1 monotherapy and ipilimumab-nivolumab, increased rates of toxicity mean ipilimumab-nivolumab is generally offered to younger fitter patients [19].

Checkpoint inhibitors are high cost drugs, with significant resource implications for clinical practice. The question of whether anti-PD-1 antibodies need to be administered chronically has been coming under increased scrutiny. Long-term follow up data from previous studies however suggest that sustained responses can be seen despite discontinuation of anti-PD-1 therapy. In the phase III study of pembrolizumab (KEYNOTE-006), the planned duration of treatment was 2 years with 19% of patients completing treatment as planned (103 of 556 patients) [20]. In this group the estimated 24-month PFS from completion of pembrolizumab was 78.4%, after a median follow up of 34.2 months [20]. In those who progressed following completion of treatment as planned (*n* = 27), 12 patients received further pembrolizumab within the study [20]. Best overall response was complete response (CR) in 3 patients, partial response (PR) in 3 patients, stable disease (SD) in 3 patients, progressive disease (PD) in 1 patient and response assessment pending in 2 patients [20]. Similar sustained responses have also been observed with nivolumab use in non-small cell lung cancer, with CheckMate 003 demonstrating 5-year PFS in more than 75% of patients who received time-limited treatment of 96 weeks [21]. These trial results therefore support discontinuation of treatment after a defined period of response without significant negative impact. Thus, many healthcare systems across the world have adopted a time-limited treatment approach of stopping treatment after 2 years in metastatic melanoma patients who remain progression-free. However, these are small numbers and so a randomised trial is warranted.

Evaluating the optimal duration of anti-PD-1 therapy remains of clinical and health-economic interest due to the impact of continued treatment on patient risk through exposure to drug toxicity [22] or face-to-face healthcare contacts as well as patient convenience and resource use. Defining optimal duration has wide implications for the use of anti-PD-1 therapy which is being rolled out across multiple tumour sites as well as being used in earlier stages of disease. The DANTE trial serves to compare stopping anti-PD-1 antibody therapy with continuing treatment in those patients who are progression-free at 1 year.

## Methods/ design

### Trial design

**Fig. 1:**
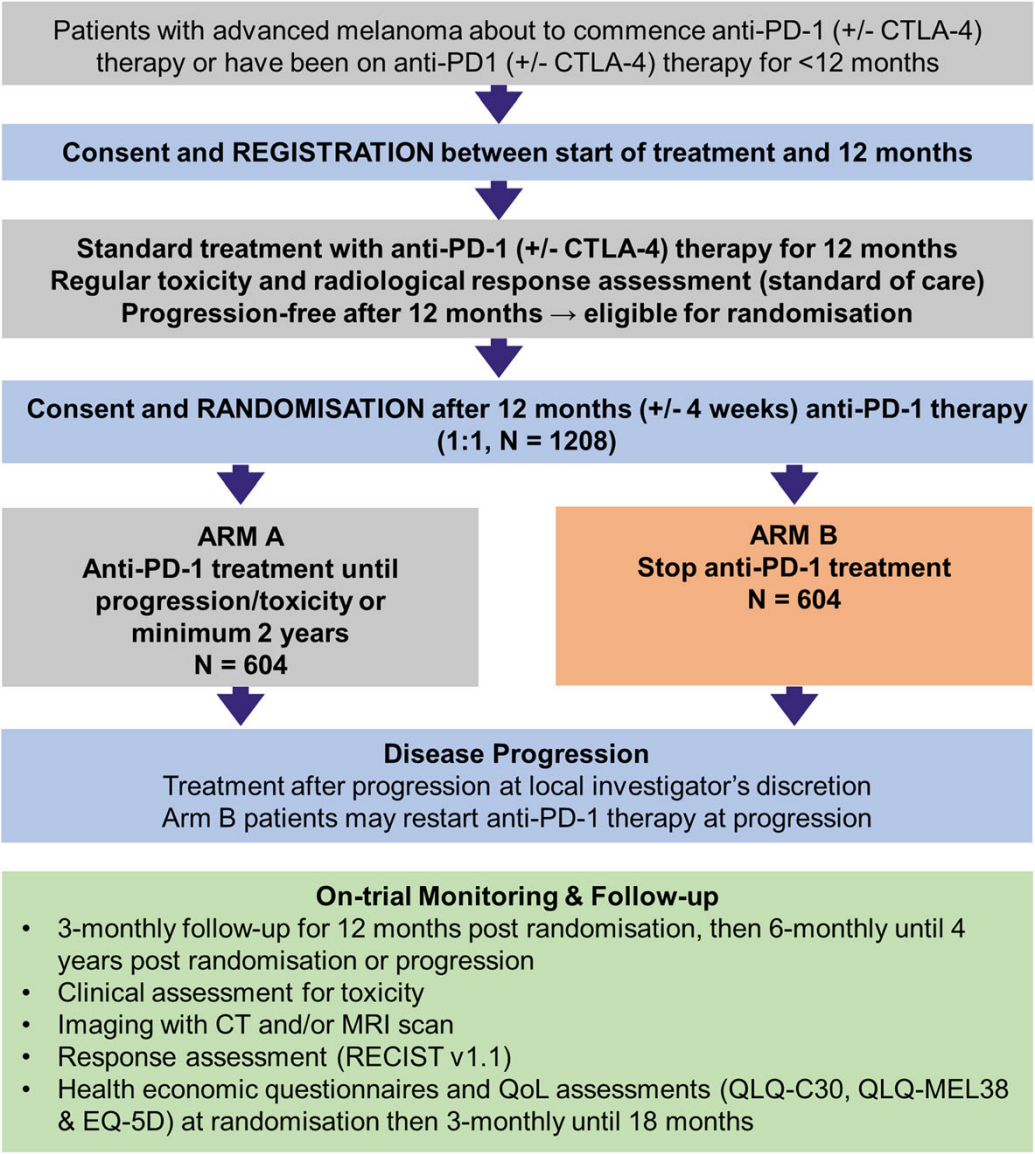
Summary of the DANTE trial

DANTE is a multicentre, randomised two-arm, parallel group, unblinded, non-inferiority phase III trial comparing time-limited treatment of 1 year of anti-PD-1 therapy (experimental arm) to the current standard duration of anti-PD-1 therapy (control arm), see Fig. 1. The ‘control’ arm of standard duration of anti-PD-1 therapy, consists of treatment until disease progression/ unacceptable toxicity, or for 2 years or more in the absence of disease progression or unacceptable toxicity, to allow generalisability to the current UK melanoma practice. The trial includes patients on nivolumab, pembrolizumab or ipilimumab-nivolumab using a licensed dosing schedule, reflecting variations across the UK and allowing greater clinician and patient choice.

The trial is multistage with planned interim assessments to ensure feasibility of recruitment (stages 1 and 2) and any lack of efficacy is identified early (stage 3), with stop-continuation rules embedded into the trial design. Provided these conditions are met, primary analysis of outcomes at 1-year post randomisation (stage 4) and long-term analysis at 4-years post randomisation (stage 5) will occur. An embedded qualitative sub study will explore patients’ perceptions about the acceptability of randomisation.

DANTE will follow a two-stage recruitment strategy with registration of patients within 1 year of starting standard of care, first line anti-PD-1 therapy and randomisation of eligible patients after 1 year of this treatment, which signifies the start of the clinical trial. The ability to register patients for trial entry prior to completion of 1 year of anti-PD-1 therapy and as such prior to consideration of randomisation introduces trial concepts early and aims to improve acceptance of subsequent randomisation. Registration also allows patients who decline randomisation to be identified for a planned qualitative assessment of reasons for patient acceptance versus decline of randomisation to help improve randomisation rates.

### Trial aims and objectives

The study aims to identify if time-limited treatment of anti-PD-1 therapies is non-inferior to standard treatment for clinical efficacy outcomes and if time-limited treatment can lead to improved quality of life and health economic outcomes due to reduction in side effects and/or reduced treatment burden.

#### Primary objective

The primary objective is to determine whether reduced duration therapy is non-inferior to standard treatment in terms of PFS.

#### Secondary objectives

Secondary objectives include quality of life (key secondary objective), overall survival, response after randomisation as defined by RECIST v1.1 criteria [23], safety and drug-induced toxicity and cost-effectiveness.

#### Interim objectives

- To demonstrate an adequate rate of patient recruitment and acceptance of randomisation (Stage 1)
- To confirm feasibility of the randomisation target across all sites (Stage 2)
- To identify early evidence of lack of efficacy or even superiority of time-limited treatment compared to standard treatment (Stage 3).

#### Long-term follow-up objectives

- To determine whether time-limited treatment is non-inferior in terms of PFS and OS at 4 years post-randomisation compared to standard treatment (Stage 4)
- To report long-term safety data of time-limited treatment and standard treatment (Stage 5).

### Trial population

DANTE aims to be representative of the current population of adult (≥18 years) patients with advanced melanoma (unresectable stage III or stage IV) on first-line anti-PD-1 therapy, either as single agent or in combination with ipilimumab. Recruitment therefore allows any patient who meets these broad criteria and is within the first 1 year of therapy to be registered.

For randomisation, which signals the start of the trial, patients must still be on anti-PD-1 therapy, be progression free by RECIST v1.1 criteria [23] at 1 year +/− 4 weeks and have been registered into the study. Patients must have ECOG performance status 0–2 and be considered fit to receive on-going anti-PD-1 treatment. Previous targeted therapy with BRAF/ MEK inhibitors is permitted. Patients treated with (neo)adjuvant therapy may be randomised if completed more than 6 months prior to start of anti-PD-1 therapy. Patients with brain metastases are eligible if asymptomatic and/or not requiring further treatment in untreated brain metastases or if radiologically stable by MRI and/or not requiring high dose corticosteroids (defined as > 10 mg per day prednisolone/ equivalents) in treated brain metastases. To remain eligible patients must not meet any of the defined exclusion criteria (severe co-morbidities including severe autoimmune disease or pneumonitis; active infection requiring systemic therapy; known active HIV, hepatitis B or hepatitis C; prior invasive cancer, excluding stage 1/2 non-melanoma skin cancer, without a disease free interval of 1-year after treatment completion; pregnancy and/or breastfeeding). Patients must agree to use adequate contraception whilst on anti-PD-1 therapy and for 6 months after completion if of reproductive potential (both male and female). All patients provide written informed consent at registration and at randomisation.

### Recruitment, randomisation and treatment allocation

Patients are recruited at UK hospitals with specialist melanoma oncology teams. Patient registration occurs at any point from the start of anti-PD-1 therapy up until 1 year (+ 4 weeks).

Randomisation occurs at 1 year (+/− 4 weeks) post start of anti-PD-1 therapy in eligible patients. Treatment allocation via a central automated 24-h system (provided by University of Leeds), using a 1:1 ratio is by minimisation, with a random element, stratifying for BRAF status (wildtype, mutant, unknown), prior use of BRAF/MEK inhibitor therapy for advanced melanoma (yes, no), prior use of (neo)adjuvant immunotherapy (yes, no), disease stage at start of anti-PD-1 treatment (unresectable stage III or stage IV disease), brain metastases (yes, no), ECOG performance status (0/1, 2), centre, treatment received within the previous 1 year (ipilimumab-nivolumab, nivolumab or pembrolizumab) and response after the first 1 year of therapy defined according to RECIST v1.1 criteria [23] (CR, PR, SD). No attempt at allocation concealment will occur due to practicalities of treatment within arms.

### Sample size

Sample size calculations are based on the primary time-point of 2-year PFS, which is equivalent to 1 year post randomisation. To inform these calculations, data from CheckMate 067 was used which showed 43% and 37% of patients were alive and progression free at 1 year and 2 years after the start of treatment respectively in the nivolumab only arm, and 50% and 43% of patients alive and progression free at 1 year and 2 years for those receiving combination therapy [24]. This correlates with a relative reduction in PFS of 14% from 1 year to 2 years. Given that 100% of the DANTE randomised population will be alive and progression free at 1 year after starting anti-PD-1 therapy (i.e. at the point of randomisation), it is estimated that approximately 86% will be alive and progression free at 2 years on standard treatment (as used in CheckMate 067 [24]). Defining non-inferiority as a reduction in 2 year PFS of no more than 6%, with 80% power and a one-sided significance level of 5%, and accounting for a 5% drop out rate, 1208 patients (604 per arm) are required in total to test for this degree of non-inferiority using a one-sided log-rank test. Consensus for the non-inferiority margin was reached after discussions within the National Cancer Research Institute (NCRI) Skin Cancer Clinical Studies Group and patient representatives.

### Baseline (pre-randomisation) investigations

All patients undergo assessment as per the current UK standard prior to the initiation of anti-PD-1 +/− CTLA-4 therapy, including cross-sectional imaging. Repeat cross-sectional imaging (within 28 days of randomisation) is undertaken and compared to the pre-treatment baseline scan by RECIST v1.1 criteria [23] to ensure progression free status at randomisation. Baseline patient-reported assessments of quality of life (QoL), using the generic EORTC QLQ-C30 questionnaire [25] and the melanoma-specific module EORTC QLQ-MEL38 [26] and health care resource use, using EQ-5D-5L (EuroQol) [27] are completed following consent but prior to randomisation.

### Intervention

At randomisation, patients allocated the control arm (standard treatment) continue on the same treatment as before and those allocated the experimental arm stop treatment. Standard treatment continues for at least 2 years in the absence of disease progression, unacceptable toxicity or patient choice to stop protocol treatment.

At progression, re-treatment with the same anti-PD-1 therapy may be considered providing strict criteria are met in line with local commissioning arrangements.

### Follow-up data collection

All data are collected on trial specific paper CRFs and submitted to the Clinical Trials Research Unit (CTRU) at the University of Leeds for management and monitoring.

Patients who continue to receive anti-PD-1 therapy within the control arm have regular toxicity assessments, at least every 12 weeks and in line with current practice at each trial site. Patients in both arms are assessed for toxicity, concomitant medications and response every 3 months (+/− 2 weeks) for 1 year post randomisation and then every 6 months (+/− 2 weeks) for up to 4 years post randomisation. Cross-sectional CT/MRI imaging is performed at these time points in accordance with standard practice, with assessment of the chest, abdomen and pelvis plus any known additional disease sites. In patients without known brain metastases, imaging of the head is performed at least 6 monthly. Toxicity is graded using CTCAE v5.0 [28] and imaging reported using RECIST v1.1 [23]. A schedule of assessment is included as supplementary material (Additional file 1: Table S1).

Patient-reported QoL [25,26] and health care resource use [27] assessments are undertaken during clinic visits or by post every 3 months (+/− 2 weeks) for 18 months post randomisation. This includes patients who have progressed during this time period where possible. The use of EQ-5D-5L (EuroQol) [27] for health care resource use assessments will allow subsequent estimation of quality adjusted life years (QALYs). Health economic data (QALYs and costs) will allow estimation of the within trial incremental cost effectiveness ratio (ICER) of the two treatment arms. Using a de novo decision-analytic model, trial outcomes will be extrapolated to generate lifetime estimates of cost-effectiveness.

All patients are followed up until 4 years post randomisation. In patients who have progressed, data on treatment administered post-progression, toxicity and survival will be collected at the planned time points using paper case report forms (CRFs) where possible and / or via routine data sources e.g. NHS Digital, Office for National Statistics or Systemic Anti-Cancer Therapy data-set.

### Safety

Selected AEs related to anti-PD-1 therapy administered before randomisation are reported for both arms. For the control arm only, all adverse reactions (ARs) related to anti-PD-1 therapy administered after randomisation are reported together with serious adverse reactions (SARs) and suspected unexpected serious adverse reactions (SUSARs) from randomisation to 5 months following the last delivered protocol treatment.

### Statistical methods and analysis

Stage 1 will take place following 9 months of recruitment. The percentage of eligible patients that agree to be randomised, from sites that have been open for 6 months, will be summarised. Pre-specified red, amber and green targets have been set to make decisions on trial continuation. The overall number of randomisations from all sites will also be presented. Stage 2 will assess the number of randomised participants from all sites within a defined 6 month period of recruitment. Trial continuation decisions will be made against a set of red, amber and green targets. At stage 1 and 2, baseline and disease characteristics will be summarised descriptively for the randomised population and all registered patients who are alive and progression-free at 12 months. Stage 3 analysis will be performed on the intention-to-treat (ITT) population and will test for early evidence of superiority (*p* < 0.005) or inferiority (*p* < 0.05) of the experimental arm against the control arm on PFS. Different alpha levels have been incorporated to reflect the relative importance of the interim analysis for superiority and inferiority claims. PFS will be investigated using Kaplan-Meier survival curves. Participants without a PFS event at the time of analysis will be censored at the date they were last known to be alive and progression-free. If the proportional hazards assumption is met, Cox’s Proportional Hazards model, adjusting for the minimisation factors, will be used to compare PFS between the treatment arms.

Analysis of non-inferiority endpoints will be performed on both the ITT and per-protocol populations; equal weighting will be given to both analyses as ITT is likely to be the least conservative approach when testing for non-inferiority. Analysis of superiority endpoints will be performed on an ITT basis with per-protocol analyses conducted if there are a sufficient number of major protocol violators; ITT analyses will however be given primacy. Hypothesis testing will be two-sided for superiority endpoints and one-sided for non-inferiority endpoints and use a 5% significance level.

Interpretation of non-inferiority for the primary endpoint of PFS will be based on the 90% (one sided type I error rate of 5%) confidence interval (CI) of the difference in PFS rates at 1 year post randomisation determined from Kaplan-Meier estimates at this time-point; the upper limit of the 90% CI will be compared with the non-inferiority margin of 6%. If it is below this margin then time-limited treatment will be declared as non-inferior to standard treatment. If the upper limit is above the non-inferiority margin then non-inferiority will not have been demonstrated. PFS and OS will be assessed using Kaplan-Meier curves. If appropriate, Cox’s Proportional Hazards model, adjusting for the minimisation factors, will be used to compare PFS and OS between the treatment groups. In addition, a sensitivity analysis of the primary endpoint will assess time to progression, where deaths without documented evidence of progression will be considered a competing-risk event. Time to progression will be investigated using cumulative incidence function curves and compared using Cox’s Proportional Hazards model if appropriate, to adjust for the minimisation factors.

The main QoL outcome of interest is the summary score of the EORTC QLQ-C30 [29]. Quality of life will be summarised at each time point using adjusted for baseline mean scores and 95% CIs. Summaries and differences between arms will be obtained and compared using a multi-level repeated measures model, allowing for time, treatment, and treatment-time interactions, and adjusting for baseline QoL and the minimisation factors (fixed effects) and participant and participant-time interaction (random effects) where appropriate, assuming missing data at random. Missing data patterns will be examined and alternative analyses using different missing data assumptions will be performed if appropriate (e.g. pattern mixture multi-level models). This methodology will be repeated to analyse scores from the EORTC QLQ-MEL38 [26].

Differences in response (defined by RECIST v1.1 criteria [23]) rates between the treatment groups will be compared using logistic regression for objective response and ordered logistic regression for best tumour response, adjusting for the minimisation factors. Sensitivity analyses will be conducted to allow for any deaths from causes other than melanoma, for whom no response status was observed. Both ITT and per-protocol analyses will be performed on the RECIST evaluable population.

Duration of response will be assessed in only those participants in the RECIST evaluable population who have a response after randomisation; deaths without documented evidence of disease progression will be considered a competing-risk event. Duration of response will be investigated using cumulative incidence function curves and compared using Cox’s Proportional Hazards model if appropriate, adjusting for the minimisation factors.

Subgroup analyses for the clinical randomisation factors and other baseline characteristics will be performed to investigate whether there is heterogeneity of treatment effect on outcomes.

Safety and toxicity will be reported descriptively. Two sets of cost-effectiveness analyses will be undertaken for the health economic evaluation: trial-based analyses, comparing costs and outcomes between time-limited and standard treatment duration up to the 18-month post-randomisation time-point; and decision-analytic model-based analyses, which will extrapolate the results of the trial over a lifetime horizon. Costs will incorporate time on treatment; and social, primary and secondary health care use costs. Unit prices will be applied to these based on standard sources (e.g. Drugs and pharmaceutical electronic market information tool [eMIT] and NHS Reference costs). QALYs will be based on the EQ-5D-5L using the approach to scoring currently preferred by NICE [30]. However, a new UK EQ-5D-5L valuation study is on-going and we will use the resulting valuation tariff if it is available at the time of analysis and providing the results are valid and robust. The trial analyses will estimate ICERs following adjustment for baseline imbalance and key minimisation factors. Parametric or non-parametric (i.e. bootstrapping) methods will be used to characterise the sampling uncertainty present with simulations plotted on a cost-effectiveness plane and cost-effectiveness acceptability curves. We will assess the type and degree of missing data and evaluate whether the assumption of missing at random (MAR) holds [31]. If that is the case, multiple imputation will be used to impute missing data. Should MAR not hold we will explore the impact of alternative assumptions [32]. We will generate a detailed health economic analysis plan (HEAP).

As the benefits of the interventions are expected to extend beyond the trial follow-up period, we will develop a decision-analytic model to estimate future costs and benefits following best practice [33]. The model type and structure will be agreed after consultation with clinical experts and patients and a review of existing models in the area.

The model will enable the calculation of discounted lifetime ICERs and estimates of net monetary benefit (NMB). We will assume a cost-effectiveness threshold of £20,000 per QALY gained. We will conduct extensive deterministic one-way and scenario sensitivity analyses. Monte Carlo simulations using draws from parameter distributions will allow a probabilistic sensitivity analysis capturing total parameter uncertainty in the model. Results from this will be presented in the form of cost-effectiveness planes, NMB distributions and cost-effectiveness acceptability frontiers [34]. Costs and benefits post 12 months will be discounted at a rate of 3.5% per annum as per NICE guidance.

### Trial organisation and administration

The Clinical Trials Unit, on behalf of the trial sponsor, ensure the trial is undertaken according to the principles of Good Clinical Practice (GCP) and in line with the relevant UK Research Governance Frameworks. Trial registration is ISRCTN15837212 and the EudraCT number is 2017–002435-42.

An independent Data Monitoring and Ethics Committee (DMEC) review the safety and the ethics of the trial with detailed unblinded interim reports submitted at least annually. The DMEC, together with the Trial Steering Committee (TSC) and the funder are responsible for enacting stop/ continuation rules during the interim stages (stages 1 to 3) if predefined targets for patient recruitment or efficacy are not met.

### Sub-studies

To inform trial recruitment both now and in the future, an integrated qualitative sub-study is included. This sub-study will explore patients’ views on decision making with regard to trial entry and subsequent randomisation. A semi-structured topic guide facilitated exploration will be undertaken for 24 patients eligible for registration (6 patients) or randomisation (18 patients, 2:1 ratio of randomised to non-randomised patients). All interviews will be audio recorded following informed consent and a thematic analysis [35] will be performed.

At recruitment, patients consent will be sought to provide access to any archival melanoma tumour samples, to be used in future translational research.

The trial protocol and this paper have been written in accordance with standard protocol items: recommendations for interventional trials (SPIRIT) guidelines [36]. A SPIRIT checklist is included as supplementary material (Additional file 2: Table S2).

## Discussion

Given the implications to both patients and health care systems across the world, there is a high international imperative to define optimal duration of therapy with anti-PD-1 antibodies. Treatment-associated burden to patients includes time, financial and potential drug induced toxicity whereas treatment-associated burden to healthcare includes capacity, drug costs and associated treatment delivery costs. DANTE hopes to answer the question as to whether continued treatment is justified accounting for these costs or if time-limited treatment can provide greater value without loss of treatment benefit.

To date, a single randomised trial, CheckMate 153, has reported only exploratory data on the optimal duration of anti-PD-1 treatment. This trial randomised patients with advanced non-small cell lung cancer to continuous and time-limited (1 year) nivolumab treatment to evaluate the incidence of high-grade select treatment AEs [37]. In this exploratory analysis, median overall survival was not reached for continuous treatment and was 23.2 months for time-limited treatment [37]. The 1 year OS rate was not statistically different between the two groups (88% for continuous treatment vs. 81% for time-limited treatment with a HR = 0.63, 95% CI 0.33–1.20) [37]. In this study of 1375 patients, only 15% (*n* = 218) remained on nivolumab at 1 year and so the number of patients eligible for randomisation was small [37]. It is also arguable that due to different immunosensitivities of different tumour types, the results are not generalisable.

Two additional clinical trials are ongoing in metastatic / unresectable melanoma to evaluate the impact of stopping treatment in patients upon achieving response: STOP-GAP and Safe Stop-T.

The Canadian STOP-GAP study (NCT02821013) [38], is a phase 3 trial which randomises eligible patients within 16 weeks of starting anti-PD-1 therapy to standard treatment of 2 years or to treatment until maximal tumour response (defined by at least 2 radiological measurements 3 months apart) with re-treatment at time of progression. STOP-GAP, designed to complete recruitment at the end of 2023, therefore primarily aims to evaluate the role of re-challenge rather than the specific question of optimal treatment duration.

The Dutch Safe Stop Trial (Safe Stop-T, NTR7502 [39]) is an observational study of the STOP & GO strategy of PD-1 blockade where treatment is stopped within 4 weeks of confirmation of radiological response (CR or PR according to RECIST v1.1 criteria [23]). Radiological monitoring continues every 12 weeks with treatment immediately re-commenced in the event of progression. The primary outcome measure is the rate of ongoing response according to RECIST v1.1 criteria [23] at 2 years after the start of PD-1 blockade. Further sub-studies include Safe Stop-QoL which evaluates the impact of this early discontinuation of treatment on patient reported disease specific and generic health-related QoL, patient productivity and informal care resource use. Safe Stop-T [39] therefore aims to provide observational data on the safety of intermittent treatment in a cohort of 200 patients rather than identify the optimal duration of treatment through a randomised trial.

A cohort study [40] in 185 patients with advanced melanoma, across various treatment centres, who electively stopped anti-PD-1 therapy in the absence of progressive disease or treatment-limiting toxicity, provides further observational data on the impact of early discontinuation of anti-PD-1 treatment. In this study patients who experienced a best objective response (BOR) of CR during treatment (117 of 185) had a shorter median duration of anti-PD-1 therapy (11 months) and were significantly less like to experience PD following treatment discontinuation compared to those who experienced a BOR of PR (HR 2.99, 95% CI 1.45–6.16) or a BOR of SD (HR 5.15, 95% CI 2.19–12.09) [40]. This supports previously observed data that durable responses can continue in patients who discontinue treatment earlier than a planned duration of 2 years (as used in KEYNOTE-006 [20] and across many worldwide healthcare settings). The observed difference in PD across the subgroups of BOR poses the question of whether response during treatment should be considered when decisions on duration of anti-PD-1 therapy are made on an individual patient basis. Future prospective studies to answer this question are however required.

The DANTE trial will help define what constitutes the optimal duration of anti-PD-1 therapy in the treatment of advanced melanoma. Designed as a prospective randomised controlled trial it will assess whether treatment should be continued for a set time and specifically whether this should for a reduced duration of 1 year. This contrasts with STOP-GAP [38] and Safe Stop-T [39] which will assess whether treatment should be continued to a set response. All three trials are however key to expanding the evidence base and ensuring patients receive the correct duration of treatment, both in terms of efficacy and costs, without being given too much or too little.

The outcomes of these studies will not only enable better patient and clinician decision making on an individual patient basis but ensure that continuation of these high cost treatments remain appropriate in a high demand, resource limited healthcare setting.

## Supplementary Information

**Additional file 1: Table S1.** Assessment schedule of the DANTE trial.

**Additional file 2: Table S2.** SPIRIT 2013 Checklist.

## Data Availability

Not applicable.

## Abbreviations

AE: Adverse event
AR: Adverse reaction
BOR: Best objective response
BRAF: A human gene that encodes a protein called B-Raf
CI: Confidence interval
CR: Complete response
CRF: Case report form
CT: Computerised tomography
CTCAE v5.0: Common Toxicity Criteria for Adverse Events version 5
CTLA-4: Cytotoxic T-lymphocyte-associated antigen 4
CTRU: Clinical Trials Research Unit
DANTE: A randomised phase III study to evaluate the Duration of ANti-PD-1 monoclonal antibody Treatment in patients with metastatic mElanoma
DMEC: Data Monitoring and Ethics Committee
ECOG: Eastern Cooperative Oncology Group
EORTC: European Organisation for Research and Treatment of Cancer
GCP: Good Clinical Practice
HEAP: Health economics analysis plan
HIV: Human immunodeficiency virus
HR: Hazard ratio
ICER: Incremental cost effectiveness ratio
ITT: Intention to treat
MAR: Missing at random
MAP: Mitogen activated protein
MEK: Mitogen activated protein kinase
MRI: Magnetic resonance imaging
NCRI: National Cancer Research Institute
NHS: National Health Service
NICE: The National Institute for Health and Care Excellence
NIHR: National Institute for Health Research
NMB: Net monetary benefit
OS: Overall survival
PD: Progressive disease
PD-1: Programmed cell death protein 1
PDL-1: Programmed cell death ligand 1
PET: Positron emission tomography
PFS: Progression-free survival
PR: Partial response
QALYs: Quality adjusted life years
QoL: Quality of life
REC: Research ethics committee
RECIST v1.1: Response Evaluation Criteria In Solid Tumours version 1.1
SAR: Serious adverse reaction
SD: Stable disease
SUSAR: Suspected unexpected serious adverse reaction
TSC: Trial Steering Committee
UK: United Kingdom
