# Supplementary material for: The DANTE trial protocol: a randomised phase III trial to evaluate the Duration of ANti-PD-1 monoclonal antibody Treatment in patients with metastatic mElanoma
Source: BMC Cancer. 2021 Jul 1;21:761. doi: 10.1186/s12885-021-08509-w (PMC8246129; doi:10.1186/s12885-021-08509-w)
Supplement: Supplementary file 1 — Additional file 1: Table S1. Assessment schedule of the DANTE trial. [file 12885_2021_8509_MOESM1_ESM.docx]

Table S1: Assessment schedule of the DANTE trial
